# Microstructure and Properties of Glass Fiber-Reinforced Polyamide/Nylon Microcellular Foamed Composites

**DOI:** 10.3390/polym12102368

**Published:** 2020-10-15

**Authors:** Xiulei Wang, Gaojian Wu, Pengcheng Xie, Xiaodong Gao, Weimin Yang

**Affiliations:** 1 College of Mechanical and Electrical Engineering, Beijing University of Chemical Technology, Beijing 100029, China; 13126623255@163.com (X.W.); wu039879202@163.com (G.W.); GXDLPH@163.com (X.G.); yangwm@mail.buct.edu.cn (W.Y.); 2 State Key Laboratory of Organic-Inorganic Composites, Beijing University of Chemical Technology, Beijing 100029, China

**Keywords:** lightweight, glass fiber reinforced nylon, supercritical fluid, microcellular foaming, injection molding

## Abstract

The automobile and aerospace industries require lightweight and high-strength structural parts. Nylon-based microcellular foamed composites have the characteristics of high strength and the advantages of being lightweight as well as having a low production cost and high product dimensional accuracy. In this work, the glass fiber-reinforced nylon foams were prepared through microcellular injection molding with supercritical fluid as the blowing agent. The tensile strength and weight loss ratio of microcellular foaming composites with various injection rates, temperatures, and volumes were investigated through orthogonal experiments. Moreover, the correlations between dielectric constant and injection volume were also studied. The results showed that the “slow–fast” injection rate, increased temperature, and injection volume were beneficial to improving the tensile strength and strength/weight ratios. Meanwhile, the dielectric constant can be decreased by building the microcellular structure in nylon, which is associated with the weight loss ratio extent closely.

## 1. Introduction

In recent years, the microcellular foaming composites are increasingly recognized as an important component for improving the structure and properties of polymeric materials due to their remarkable effects such as lightweight [1,2,3] and low dielectric constant [4,5]. The microcellular foaming process can generate large numerous of micron-sized cells inside the polymer matrix, forming a unique dense surface and core foamed structure [6,7,8]. This structure could reduce the weight of the material, and it endows the material with higher toughness, impact strength, and dimensional accuracy [9,10,11,12,13], which is meaningful to the requirement of lightweight. Nylon (PA)-based composites are an important engineering material with excellent mechanical properties, thermal stability, and processability [14], particularly glass fiber-reinforced nylon (GF/PA). Adding the glass fiber into the PA matrix could achieve increased load-bearing capacity and mechanical properties because the fibers retard the rapid growth of cracks [15,16]. The interface of each fiber and the matrix can refine the crack and cause it to propagate in multiple directions, thus extending the propagation length. The fracture in the GF/PA changes from a single-matrix fracture to a combined of matrix fracture, fiber pullout, and fiber broken, which helps increase the mechanical toughness [17,18,19]. GF/PA microcellular foamed composites have the characteristics of high strength and the advantages of being lightweight as well as having a low production cost and high product dimensional accuracy, because the addition of fibers could promote nucleation, refining the cell structure [20]. Therefore, GF/PA has gradually expanded from the initial interior parts to structural parts such as engines, bodies, and bumpers for vehicle application. In addition, GF/PA microcellular foamed composite will also have a positive impact on the development of automobile and aerospace structural parts.

At present, researchers have focused on studying PA-based foamed composites. Wang et al. [21] prepared GF/PA6 composites by chemical foaming injection molding and discussed the influence of foaming agent masterbatch on the cell morphology and mechanical properties. However, compared with the chemical foaming method, supercritical fluid foaming has huge potential in the field of lightweight due to the advantages of being environmentally friendly, controllable, and having no chemical residual process [22,23]. Wang et al. [24] and Hwang et al. [25] prepared PA6 micro-sized cells materials by the supercritical fluid physical foaming method and discussed the mechanical properties of the products. Valentina Volpe et al. [26] studied the effect of gas injection pressure and thickness of the parts on the mechanical properties of GF/PA 66 microcellular foams. As a result, a high gas injection pressure and a large thickness of the parts are adopted to obtain homogeneous foamed parts with good mechanical properties. Roch et al. [27] show the relationship between different density reductions and bending stiffness of GF/PA6 foams with breathing mold technology. All studies have shown that foaming can increase the specific strength of composites to a certain extent. However, there are few studies on the effects of critical injection process parameters on the properties of GF/PA microcellular foams based on the supercritical fluid physical foaming method.

In this paper, for the first time, we comprehensively studied the microstructure and strength/weight loss rate of GF/PA 610 microcellular foams with various injection rates, temperatures, and injection volumes based on supercritical fluid microcellular foam injection molding. Especially, we analyzed the correlation between microstructure and foam strength in detail in the study. According to the conclusions of this paper, the mechanical properties of GF/PA microcellular foams are adjusted by refining the microstructure to fulfill product requirements and to be more lightweight. In addition, structural parts such as integrated circuit shells and aircraft fuselages must have low dielectric constant characteristics to avoid excessive current passing through composites and damaging internal products at the working surface [28]. Therefore, we have further studied the correlation between dielectric constant and weight loss rate of the foamed material. The work makes up for the deficiency of the current research data on the properties of GF/PA 610 microcellular foams and provides a useful reference for the comprehensive evaluation of GF/PA microcellular foams.

## 2. Experimental

### 2.1. Materials

PA 610 (3RILSAN SMVO-F, Arkema (Suzhou) Polymer Materials Co., Ltd., Feng Huang Town, China) with the melt point of 222.5 °C and the relative viscosity of 2.56 was used in the experiment. GF/PA 610 composite material (SLL/G30) containing 30 wt % glass fiber was prepared by the Aerospace Research Institute of Materials and Processing Technology, Beijing, China. The supercritical nitrogen with 99.99% purity was purchased from Beijing Li Synthetic Gas Co., Ltd. (Beijing, China).

### 2.2. Experiment Procedure

**Figure 1:**
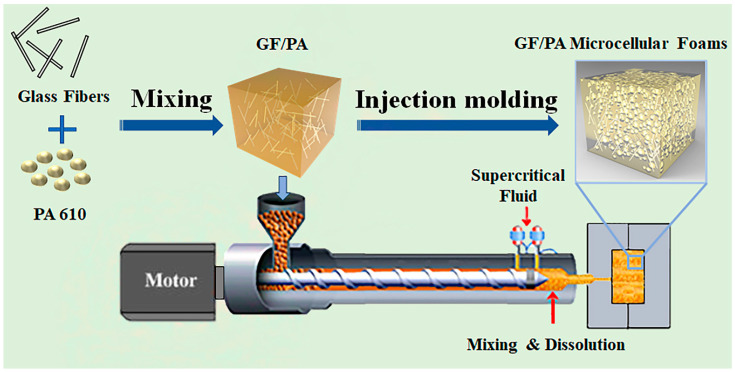
The molding flow chart of glass fiber-reinforced nylon (GF/PA) microcellular foams.

Figure 1 illustrates the GF/PA microcellular foams molding process for this experiment.

### 2.3. Preparation of GF/PA Microcellular Foams

A 160-ton modified injection molding machine (MA1600IIS, Haitian, China) equipped with a supercritical fluid injection unit was used to prepare GF/PA microcellular foams. The gas compression and control equipment were supplied by Beijing Chn-top Moldplastech Co. Ltd. (Beijing, China).

The PA pellets were dried for at least 6 h at 80 °C in the dehumidifying dryer to remove moisture before use. The microcellular foamed GF/PA 610 composites were manufactured based on the orthogonal experimental method by controlling the injection volume, temperature, and injection rate, as shown in Table 1. According to the ASTM D638-03 standard, the specimen for tensile strength test was prepared, and the 2 mm thickness disc samples were prepared for the characterization of dielectric properties. Among them, the trial with an injection rate of 100-100% (B5) was unable to prepare a complete sample because the exorbitant pressure drop resulted in a large amount of gas escaping.

### 2.4. Characterization

Microstructural and macroscopic properties are important indicators to evaluate the quality of microcellular foam products [6]. In this work, a scanning electron microscope (Quanta FEG 650, FEI Company, Hillsboro, OR, USA) was used to observe the micro-sized cells structure. The tensile strength of the standard specimen was measured using an electronic universal testing machine (CMT5205, Meters Industrial Systems Co., Ltd., Suzhou, China). The weight of the sample was measured using an electronic balance (ME3002, METTLER TOLEDO Instruments (Shanghai) Co., Ltd., Shanghai, China). The dielectric constant was evaluated in the X-band (8.2–12.4 GHz) using the rectangular waveguide method. The dielectric constant measurement and analysis instrument were modified by the Aerospace Research Institute of Materials and Processing Technology, Beijing, China.

To compare the mechanical properties of the foamed GF/PA composite with the mechanical properties of un-foamed products, this paper defines the “tensile strength/weight ratio N” with reference to the concept of specific strength [29]. The N was used to analyze the tensile properties of the product, and it was calculated as shown in Equation (1). The larger the N value, the higher the tensile strength of the microcellular foam product with the same density or weight loss ratio.

(1) $$N=\frac{1-\left[\left(\sigma_{0}-\sigma \right)÷\sigma_{0}\right]}{1-\left[\left(m_{0}-m\right)÷m_{0}\right]}$$

where “$\sigma_{0}$” is the tensile strength of unfoamed products, “σ” is the tensile strength of foamed products, and “$m_{0}$” and “m” are the quality of unfoamed products and foamed products.

The ImageJ software was used to analyze the cell information of the microcellular foams based on SEM images. According to the number of closed cells counted in a unit area (1 cm^2^), we use the following formula to calculate the cell density N_0_ (cells/cm^3^) [8]:

(2) $$N_{0}={\left(n/A\right)}^{3/2}\times \left(1/\emptyset \right)$$

where “*n*” is the number of cells in a unit area (1 cm^2^), “*A*” is the average unit area (1 cm^2^) of the micrograph, and “∅” is the weight retention rate.

## 3. Results and Discussion

### 3.1. Cellular Morphology

**Figure 2:**
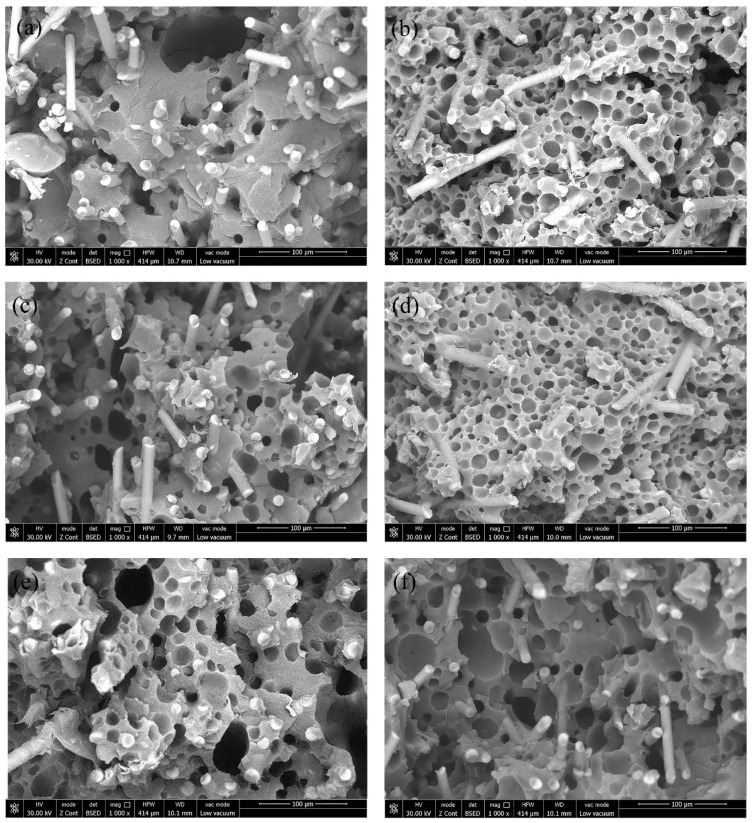
Microcellular structure of GF/PA foams at different factor levels: (**a**) The injection rate is 80–80% in the case of A3, (**b**) the injection rate is 50–80% in the case of A4, (**c**) the temperature is 250 °C in the case of B2, (**d**) the temperature is 270 °C in the case of B4, (**e**) the injection volume is 35.2 cm^3^ in the case of C3, and (**f**) the injection volume is 40.2 cm^3^ in the case of C5.

The cell structure is a key factor affecting the final properties of microcellular foamed composites [30]. Figure 2 shows the comparison of SEM photos of GF/PA microcellular foam products with different injection rates, temperatures, and injection volumes. Glass fiber and micro-sized cells were uniformly distributed in foamed composite without obvious manufacturing defects, as shown in Figure 2. The actual structure in the figure is similar to the theoretical deduction structure [22]; that is, the injection rate, temperature, and injection volume are key points that affect the cell structure. Therefore, the samples could be used to evaluate the influence of process parameters on product performance.

**Figure 3:**
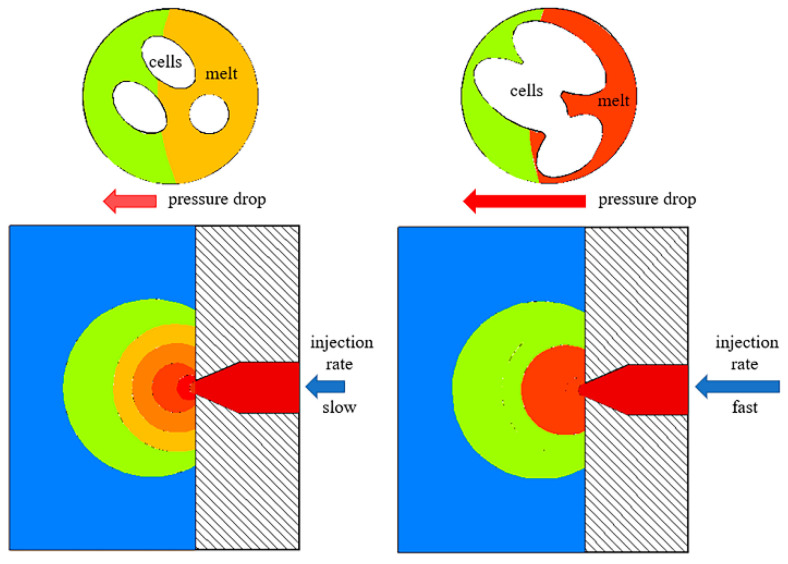
Schematic diagram of the correlation between the initial injection rate and the cell structure in the melt front.

The injection rate is closely related to the cell nucleation during the microcellular foam injection molding process. According to classical theory, the increased injection rate could enhance the melt pressure drop rate; thereby, more nucleation points were formed inside the matrix [31]. However, as shown in Figure 2a,b, the high injection rate (A3) results in the formation of larger cells, smaller cell numbers, and irregular cell structure. The number of cells in the composite shown in Figure 2a is about 5.35 × 10^7^ cells/cm^3^, and the number of cells in the composite shown in Figure 2b is about 13.58 × 10^7^ cells/cm^3^. Furthermore, the finer cell structure was achieved with the condition of the lower injection rate (A4) in the initial stage, which is that the pressure in the mold cavity (outside the melt front) is close to atmospheric pressure, while the pressure inside the melt is the injection pressure in the initial injection stage. The cell wall rupture that was caused by the melt strength decreased as did the strain hardening [32] due to the extreme pressure difference and pressure drop between the inside and the outside of the melt front when the injection rate is extremely high in the initial stage. Then, the cells of the melt front will merge and collapse as the cell wall ruptures. However, decreasing the injection rate in the initial stage could reduce the pressure difference between the melt front and the cavity, avoid the melt strength decrease and strain hardening for the melt front; then, the finer cell structure was achieved. This paper has illustrated the correlation between the initial injection rate and the cell structure at the melt front, as shown in Figure 3. The mold cavity pressure gradually increases during the filling process. The increased injection rate in the second stage could increase the rate of the melt pressure drop and cell nucleation, satisfying the classical nucleation theory. Therefore, the “slow–fast” injection rate could lead to a finer cell structure and avoid cells merging and collapsing when molding GF/PA foamed composites.

According to the classical nucleation theory, the increased melt temperature was also beneficial to improve the cell nucleation rate and thus achieve a finer cell structure [33,34]. As shown in Figure 2c,d, the cell structure was improved when the temperature was increased by 20 °C. The temperature increased is conducive to the mixture of the supercritical fluid and the polymer melt, and it is conducive to filling the cavity with the GF/PA. The temperature increase could slow down the cooling rate of the melt and help more bubble nuclei overcome the energy barrier to grow up and form cells, thereby increasing the number of cells and reducing their size [35]. The number of cells in the composite shown in Figure 2c is about 4.52 × 10^7^ cells/cm^3^, and the number of cells in the composite shown in Figure 2d is about 17.20 × 10^7^ cells/cm^3^. Therefore, the paper proves that the temperature increase within the range that does not decompose the polymer is also beneficial to refining the cell structure of the GF/PA 610 foams.

The injection volume had a direct impact on the cell growth space, which is associated with the final structure. As shown in Figure 2e,f, the foams at small injection volume had a large amount of cell collapse and a greater number of cells. The number of cells in the composite shown in Figure 2e is about 6.63 × 10^7^ cells/cm^3^, and the number of cells in the composite shown in Figure 2f is about 2.78 × 10^7^ cells/cm^3^. As a result, the injection volume decreased equal to the increased cell growth space in the same cavity volume, thus resulting in an increased risk of cell wall rupture after the increased cells volume, the thinner cell walls, and the decreased strength of the cell walls. The injection volume could be adjusted by the pressure transfer point in the injection molding process. The injection volume decrease had an adverse effect on the foam performance.

### 3.2. Tensile Strength/Weight Ratios

The tensile strength and weight loss ratio are important criteria for evaluating the macroscopic properties of microcellular foams. The tensile strength determines the use range of the GF/PA foams, and the weight loss ratio reflects the lightweight effect of the GF/PA foams. More important, the tensile strength/weight ratio comprehensively determines the degree of performance of the GF/PA foams with the same density.

**Figure 4:**
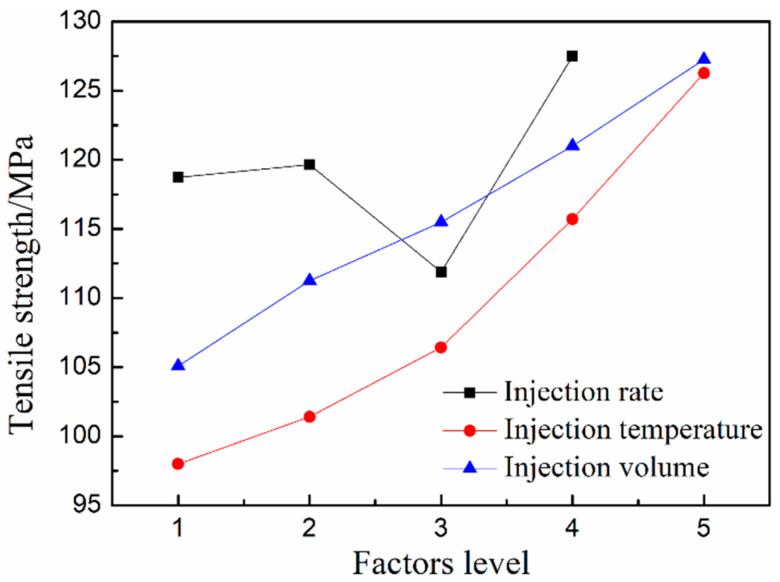
Tensile strength of GF/PA foams at different factor levels.

Figure 4 shows the tensile strength of GF/PA microcellular composites with different injection rates, temperatures, and injection volumes. The tensile strength of foams shows an overall upward trend as the injection rate increased. However, the high speed (A3: 80-80) in the entire stage led to the decreased tensile strength, which was related to the escaping gas and cell collapse in the melt front for the excessive pressure difference in the initial stage. As a result, the tensile strength was significantly improved without cavity pressure control after reducing the initial injection rate. The results prove that the “slow–fast” injection rate is beneficial to increase the tensile strength of the foamed composite. As shown in Figure 4, the tensile strength has a linear positive correlation with the injection volume and temperature, which is that the temperature increase could improve the nucleation rate, promote melt filling into the cavity, optimize the micro-sized cells structure, and thus enhance the tensile strength. Furthermore, the injection volume increase contributed to reducing the porosity of the product and avoiding the phenomenon of cell collapse. The uneven cell structure in the foamed material caused by cell collapse will lead to stress concentration and reduce the tensile strength of the material [35]. In addition, the positive correlation coefficient (slope) controlled by the temperature is significantly bigger than the positive correlation coefficient controlled by the injection volume. Therefore, the temperature increase is more conducive to rapidly increasing the tensile strength of GF/PA foams than the injection volume increase when there is only one variable.

**Figure 5:**
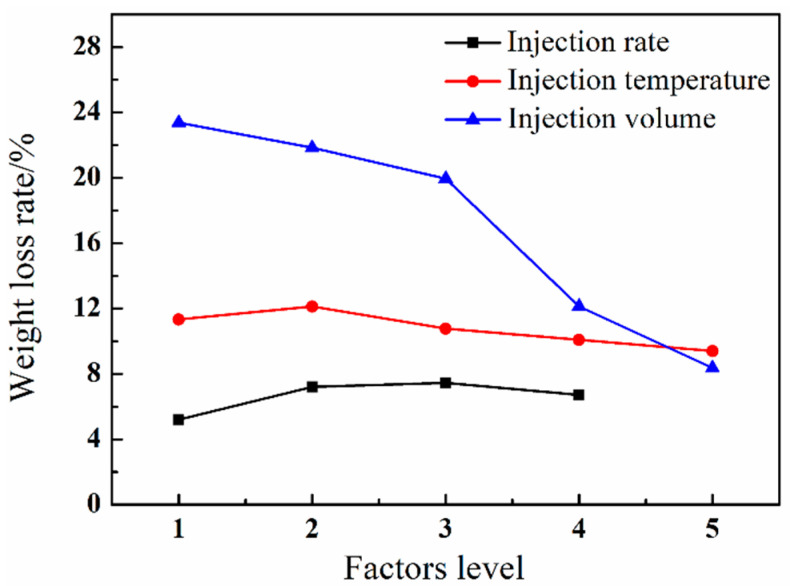
The weight loss ratio of GF/PA foams at different factor levels.

Figure 5 shows the comparison of the weight loss ratio of GF/PA microcellular foams with different injection rates, temperatures, and injection volumes. The injection volume had the more significant impact on the weight loss ratio of the product than the injection rate and temperature; that is, the injection volume has the more obvious impact on lightweight. The injection volume increase was equivalent to the increase in the volume of melt entering the cavity; thereby, the growth space of the cells reduced, and the weight loss ratio reduced in the same cavity volume. The temperature and injection rate had limited impact on the weight loss ratio compared with the effect of injection volume, as shown in Figure 5. The temperature and injection rate indirectly affected the weight loss ratio through single-phase melt formation and the cavity-filling process. Therefore, decreasing the injection volume was the main measure to enhance the lightweight of foamed composites.

**Figure 6:**
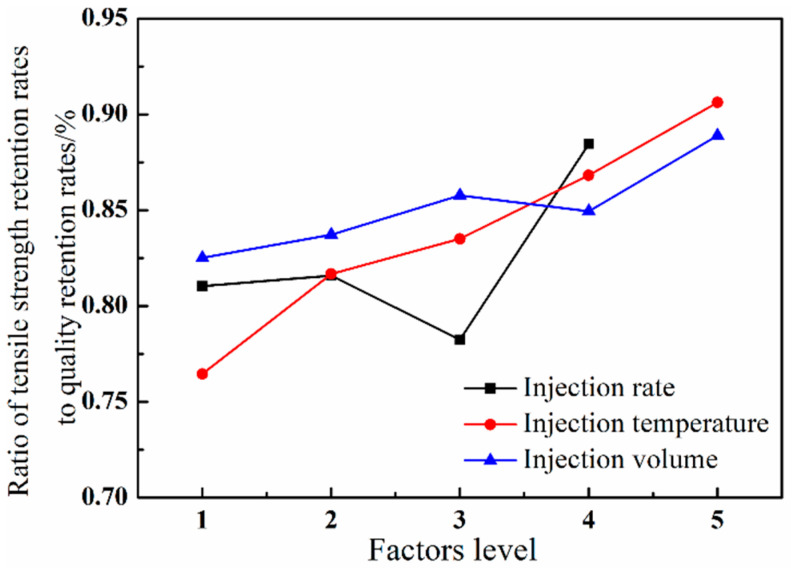
The ratio of tensile strength retention rates to quality retention rates of GF/PA foams at different factor levels.

Figure 6 shows the tensile strength/weight ratio (N) of GF/PA microcellular foams with different injection rates, temperatures, and injection volumes. The trends of the curves in Figure 6 and Figure 4 are similar, and the slope of the curve in Figure 6 is obviously affected by the slope of the curve in Figure 5. The injection rate and temperature had little effect (slope ≈ 0) on the weight change of microcellular foams in Figure 5. Therefore, the N value trend was associated with tensile strength closely. For example, the increased temperature was beneficial to promote the tensile strength within the temperature range of 40 °C, which also made the N value increase with the injection temperature. Furthermore, the curve of the N value decreased as a result of the curve of the tensile strength decreasing when the injection rate is at Level 3 (80-80). As a result, the tensile strength is a decisive factor affecting the N value when the variables are the temperature and injection rate. Moreover, the N value at different injection rate levels was basically consistent with the trend of tensile strength, which further verified that reducing the initial injection rate at high-speed levels could decrease the loss of tensile strength with the same GF/PA composite density, indirectly indicating that the finer microcellular cell structure could increase the strength of the foams.

The injection volume had a significant effect on the weight loss ratio compared with the injection rate and injection temperature. As shown in Figure 6, the curve growth trend of the N value controlled by the injection volume was obviously slower than the curve growth trend of the tensile strength controlled by the injection volume in Figure 4 due to the influence of the weight loss ratio. In other words, the weight loss ratio increased with the decreased injection volume, which also led to the increased risk of cell collapse; then, that decreased the tensile strength. That is, the increasing extent of N value was lower than the tensile strength due to the weight loss ratio increasing faster when comprehensively considering the effect of the weight loss ratio. Therefore, the single change of tensile strength could not reflect the effect of injection volume on the GF/PA microcellular foams. In addition to considering the lightweight effect, it is necessary to adjust the weight loss ratio in accordance with the requirements of mechanical properties to obtain a higher N value in actual process applications. As shown in Figure 6, the slope controlled by the injection volume was lower than the slope controlled by the injection rate and temperature. Therefore, the tensile strength decrease of GF/PA foams caused by the decreased injection volume could be compensated by using the “slow–fast” injection rate or improving the temperature.

### 3.3. Dielectric Constant

**Figure 7:**
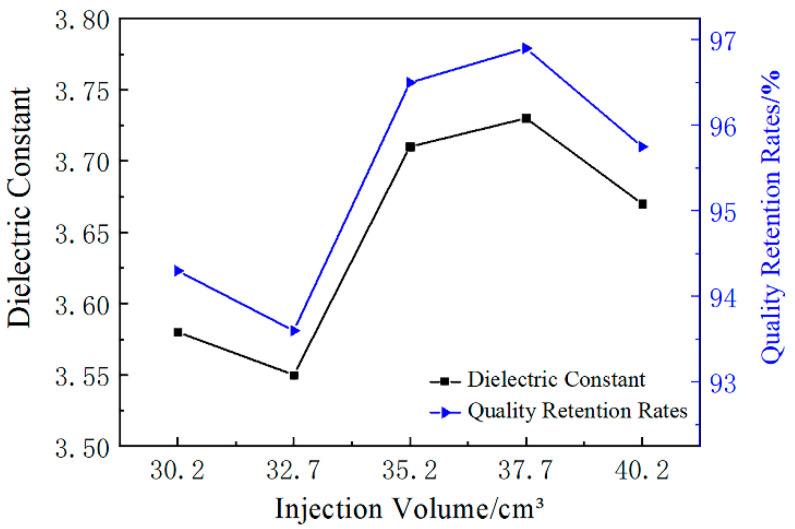
The dielectric constant and quality retention rates of GF/PA foams with different injection volume levels.

The foaming process is a common method to make foamed materials with low dielectric constants [4,5]. Figure 7 shows the correlation between dielectric constant and weight loss ratio of GF/PA microcellular foaming composites with different injection volumes. The weight retention rate increased and the porosity gradually decreased as the injection volume increased. Figure 7 shows that the trend of dielectric constant is highly similar to the trend of weight retention rate. For example, the curve of the weight retention rate in the case of 35.2 cm^3^ exhibited oscillations on account of the fluctuation of gas injection amount. Furthermore, the curve of the dielectric constant also exhibited oscillations in the case of 35.2 cm^3^, which proves that the porosity of foams is a key factor affecting the dielectric constant [36]—that is, the porosity increase could effectively reduce the dielectric constant compared with GF/PA 610 with a dielectric constant above 3.8 produced by the traditional injection molding process. Therefore, the dielectric constant of foams could be changed by adjusting the injection volume, which is highly related to the porosity.

At the same time, the paper inferred that the local area of the foams was also consistent, indicating that the local porosity of the foams was a key factor affecting the dielectric constant of the corresponding position. Therefore, the injection rate and temperature closely related to the local porosity and cell structure will also affect the dielectric constant of the foamed composites. The influence of injection rate and temperature on the dielectric coefficient of the material will be small on average, but the influence on the dielectric uniformity of different positions of the product will be significant. That is, the “slow–fast” injection rate and the temperature increase could optimize the uniformity of the product’s dielectric constant.

## 4. Conclusions

The injection rate, temperature, and volume are key factors affecting the cellular morphology and macroscopic properties of GF/PA microcellular foams. This paper uses the N value to comprehensively reflect the effects of various parameters on the macro-mechanical properties. Increasing the injection rate could enhance the pressure drop rate and cell nucleation, but an excessively high initial injection rate will cause gas overflow and the cells of the melt front to merge and collapse. Therefore, a reduced initial injection rate and “slow–fast” injection rate could diminish the pressure difference between the melt front and the mold cavity, which improved the cell structure and tensile strength. The injection temperature had an effect on the formation of single-phase melt and the cell nucleation rate. The temperature increase could improve the nucleation rate, promote melt filling into the cavity, optimize the micro-sized cells structure, and thus enhance the tensile strength. The injection volume was the direct factor influencing the weight loss ratio and porosity of GF/PA microcellular foams. The injection volume decrease contributed to improving the lightweight effect of the product rapidly. However, this will reduce the tensile strength of the product. The temperature increase is more conducive than the injection volume increase to rapidly increasing the tensile strength of GF/PA foams when there is only one variable. In order to achieve lightweight effects, the tensile strength decrease of GF/PA foams caused by the decreased injection volume could be compensated by using the “slow–fast” injection rate or improving the temperature. Furthermore, the porosity of foams is a key factor affecting the dielectric constant. The injection volume decrease could increase the porosity and reduce the dielectric constant of the foamed composites, and the “slow–fast” injection rate and the temperature increase could optimize the uniformity of the product’s dielectric constant.

## Figures and Tables

**Table 1:** The experiment factors and levels.

Level	Factor A Injection Rate/%	Factor B Temperature/°C	Factor C Injection Volume/cm^3^
1	20-20	240	30.2
2	50-50	250	32.7
3	80-80	260	35.2
4	50-80	270	37.7
5	100-100	280	40.2

Notes: The injection rate is divided into two stages, for example, “50-80” refers to the first half of the volume (0–50%) being injected at 50% of maximum injection rate, while the second half of the volume (50–100%) is injected at 80%. The maximum injection rate is 148 cm^3^/s. The temperature refers to the maximum barrel temperature. The injection volume was determined by the actual screw injection distance, and the screw diameter of the injection molding machine is 40 mm.
